# Precision redox medicine in andrology: Moving beyond empiric antioxidant use for evidence-based male infertility care

**DOI:** 10.17179/excli2026-9536

**Published:** 2026-06-26

**Authors:** Sulagna Dutta, Pallav Sengupta

**Affiliations:** 1 Basic Medical Sciences Department, College of Medicine, Ajman University, Ajman, United Arab Emirates; 2 Centre of Medical and Bio-allied Health Sciences Research, Ajman University, Ajman, United Arab Emirates; 3 Department of Biomedical Sciences, College of Medicine, Gulf Medical University, Ajman, United Arab Emirates

## ⁯⁯⁯

Oxidative stress (OS) is now firmly embedded in male infertility research, yet the field still behaves as if 'less reactive oxygen species (ROS) is always better'. That assumption is increasingly untenable. Spermatozoa are proficient ROS generators and tightly regulated ROS pulses participate in capacitation-associated signaling, including tyrosine phosphorylation cascades and membrane remodeling, and undue fall in antioxidant levels can suppress these physiological events (Aitken, 2017[1]).

Two evidence streams should force a recalibration. First, mechanistic work over two decades has supported a biphasic concept, i.e. ROS are required within a constrained range for normal sperm function, while higher ROS burdens drive lipid peroxidation mitochondrial dysfunction and sperm DNA fragmentation (SDF) (Aitken, 2017[1]). Second, clinical trial evidence has not delivered consistent reproductive endpoints with empiric antioxidant supplementation. A large, contemporary randomized clinical trial in fertility-care-seeking men reported no significant improvement in ongoing pregnancy with an antioxidant supplement versus placebo, despite the popularity of such interventions (de Ligny et al., 2025[3]). Meta-analytic syntheses remain heterogeneous, often showing improvements in selected semen metrics or pregnancy but weak/insufficient evidence for live birth and substantial between-study variability, partly reflecting non-phenotyped enrollment and non-standardized redox endpoints (Dutta et al., 2019[4]).

What has been under-addressed in Andrology is the mirror image of OS, i.e. reductive stress (RS), a pathological shift toward an overly reduced intracellular milieu (elevated NADH/NAD^+^, NADPH/NADP^+^, and/or GSH/GSSG), capable of disrupting redox-dependent signaling and proteostasis. RS is well-recognized in cardiometabolic biology, importantly, modern molecular data show that sustained NRF2 activation can induce NADH-driven RS (Weiss-Sadan et al., 2023[5]). In reproductive medicine, RS is rarely measured, rarely discussed with patients, and almost never used to stratify antioxidant trials, despite the biologic plausibility that 'antioxidant excess' can push susceptible men past a functional redox set-point and blunt ROS-dependent capacitation.

Evidence from oxidation-reduction potential (ORP) data consistently report higher seminal ORP values in infertile men compared with fertile controls, with significant correlations to reduced motility, abnormal morphology, and increased SDF (Christoforaki et al., 2025[2]). At the opposite extreme, emerging mechanistic and clinical data indicate that excessive antioxidant tone or sustained NRF2 signaling can shift intracellular redox balance toward reductive dominance, disrupting mitochondrial electron flow and ROS-mediated signaling required for fertilization (Aitken, 2017[1]; Weiss-Sadan et al., 2023[5]). These findings support a U-shaped relationship between redox tone and male fertility, with adverse functional consequences at both oxidative and reductive extremes.

Therapeutic benefit appears contingent on whether interventions measurably move redox biomarkers toward a physiological range, i.e. indiscriminate antioxidant use risks biological non-response or paradoxical deterioration. The inconsistent effects of antioxidant supplementation on pregnancy and live birth in randomized studies underscore the limitations of enrolling unstratified populations without baseline redox characterization (de Ligny et al., 2025[3]).

Clinically, these data argue for redox-aware practice, i.e. baseline assessment using ORP, integration with SDF, and objective monitoring of redox movement during treatment (Supplementary Table 1). Patient counseling must also evolve, as accumulating evidence indicates that a 'more reducing' intracellular state is not inherently safer when redox cycling itself governs sperm signaling (Aitken, 2017[1]; Weiss-Sadan et al., 2023[5]). Recognizing OS and RS as interlinked components of a single measurable continuum offers a rational path toward individualized, evidence-based reproductive care.

## Notes

Sulagna Dutta and Pallav Sengupta contributed equally as first author.

## Declaration

### Ethics approval and consent to participate

Not applicable. This letter is based solely on critical interpretation of previously published literature and does not involve human participants, animals, or identifiable personal data.

### Availability of data and materials

Not applicable. No new data were generated or analyzed for this correspondence.

### Funding sources

The authors received no specific funding for this work.

### Conflict of interest

The authors declare that they have no known competing financial interests or personal relationships that could have appeared to influence the work reported in this paper.

### Artificial Intelligence (AI) - assisted technology

During the preparation of this article, the authors used ChatGPT (version 5.2) to improve the English language quality. Following the use of this tool, the authors reviewed and edited the content as necessary and take full responsibility for the integrity, accuracy, and originality of the published work.

## Supplementary Material

Supplementary information
