# Supplementary material for: Precision redox medicine in andrology: Moving beyond empiric antioxidant use for evidence-based male infertility care
Source: EXCLI J. 2026 Jun 26;25:954–6. doi: 10.17179/excli2026-9536 (PMC13365111; doi:10.17179/excli2026-9536)
Supplement: Supplementary information [file EXCLI-25-954-s-001.pdf]

EXCLI Journal 2026;25:954-956 – ISSN 1611-2156
Received: May 05, 2026, accepted: June 04, 2026, published: June 26, 2026

Supplementary information to:

Letter to the editor:

PRECISION REDOX MEDICINE IN ANDROLOGY: MOVING BEYOND EMPIRIC ANTIOXIDANT USE FOR EVIDENCE-BASED MALE INFERTILITY CARE

Sulagna Dutta[1,2†], Pallav Sengupta[3†*]

[1] Basic Medical Sciences Department, College of Medicine, Ajman University, Ajman, United Arab Emirates
[2] Centre of Medical and Bio-allied Health Sciences Research, Ajman University, Ajman, United Arab Emirates
[3] Department of Biomedical Sciences, College of Medicine, Gulf Medical University, Ajman, United Arab Emirates

† Both the authors have contributed equally to the manuscript.

* **Corresponding author:** Pallav Sengupta, PhD, Department of Biomedical Sciences, College of Medicine, Gulf Medical University, Ajman, United Arab Emirates; Phone: +971503083217; E-mail: pallav_cu@yahoo.com

https://dx.doi.org/10.17179/excli2026-9536

This is an Open Access article distributed under the terms of the Creative Commons Attribution License (https://creativecommons.org/licenses/by/4.0/).

EXCLI Journal 2026;25:954-956 – ISSN 1611-2156
Received: May 05, 2026, accepted: June 04, 2026, published: June 26, 2026

Supplementary Table 1: Minimal phenotyping and trial endpoints for 'redox set-point'-guided antioxidant studies in male infertility

Domain	Minimum reporting elements (baseline)	Intervention monitoring (timing)	Endpoints (mechanistic + clinical)	Response categories (predefined)	Safety/stop criteria (avoid RS overshoot)
**Population & baseline redox phenotype**	Male infertility context (primary/secondary; idiopathic vs identified factor); abstinence duration; fever/acute illness history; major confounders (smoking, BMI/obesity, varicocele, metabolic disease, medications, current antioxidant use). Baseline redox status anchored to at least one 'integrated' seminal redox metric (e.g., ORP) and a conventional semen profile (concentration, motility, morphology)	Confirm no new antioxidant initiation outside protocol; document adherence and concurrent supplements	Baseline stratification into OS-dominant vs. 'low-ROS/possible RS susceptibility' strata (see categories)	OS-dominant: elevated ORP with impaired semen parameters<br>Indeterminate/mixed: modest ORP elevation with variable semen metrics<br>Low-ROS/RS-susceptible: low ORP/strong reducing tone *plus* poor functional signs (e.g., low capacitation proxy)	Exclude/hold if participant is already on high-dose multi-antioxidants and unwilling to wash out; require standardized washout period when feasible
**Oxidative stress (OS) metrics**	At least one integrated OS metric (preferred: seminal ORP) because ORP has been consistently associated with infertility, reduced motility/morphology, and higher SDF in recent datasets/meta-analyses referenced in the manuscript	Repeat ORP (or equivalent OS metric) at ~4–6 weeks and ~10–12 weeks (to capture sperm maturation cycle), plus at end-of-treatment	Primary mechanistic endpoint: change in ORP and directionality toward the physiological range; correlate with SDF and motility change	Responder (OS-corrector): ORP decreases toward physiologic window with parallel improvement in SDF and/or motility<br>Non-responder: ORP unchanged with no functional gain	Stop/step-down if ORP drops sharply below baseline with concurrent reduction in capacitation proxy or motility (suggesting over-quenching)
**Reductive stress (RS) proxies**	At least one reducing-tone proxy, ideally from semen or sperm fraction: GSH/GSSG, NADH/NAD$^+$, NADPH/NADP$^+$, and/or a redox-buffering signature consistent with "over-reduction"<br>Document "high antioxidant exposure" history	Repeat RS proxy at same timepoints as ORP; monitor symptom/lab signals compatible with over-reduction or impaired sperm signaling	Evidence of redox overshoot: rising reducing ratios with stable/worsening functional readouts	RS-shifted: ORP decreases but reducing-tone proxy rises and capacitation proxy worsens/plateaus	Protocol-defined discontinuation if reducing-tone proxy crosses a pre-set threshold *and* functional endpoints worsen (e.g., motility or capacitation proxy declines)

EXCLI Journal 2026;25:954-956 – ISSN 1611-2156
Received: May 05, 2026, accepted: June 04, 2026, published: June 26, 2026

Domain	Minimum reporting elements (baseline)	Intervention monitoring (timing)	Endpoints (mechanistic + clinical)	Response categories (predefined)	Safety/stop criteria (avoid RS overshoot)
**Sperm DNA fragmentation (SDF)**	Baseline SDF (assay specified, threshold pre-declared); SDF is central in the manuscript as a downstream consequence of high ROS burden and as a clinically relevant readout	Repeat SDF at end-of-treatment; optional interim at 6 weeks in high-baseline SDF	Co-primary mechanistic endpoint: ΔSDF (absolute and relative); assess concordance with ORP shift	Genomic responder: SDF decreases with ORP movement toward physiologic window<br>Discordant: ORP improves without SDF improvement (suggests alternate drivers)	If SDF worsens while ORP falls markedly, consider RS overshoot or other confounders; pause therapy and reassess
**Capacitation proxy (redox-dependent signaling)**	At least one capacitation-associated signaling proxy because the manuscript emphasizes that physiological ROS pulses support capacitation signaling and excessive quenching can suppress it<br>Examples: global tyrosine phosphorylation pattern post-capacitation stimulus; membrane cholesterol efflux marker; hyperactivation metric	Repeat at baseline and ~6–12 weeks or end-of-treatment; standardize incubation conditions	Mechanistic validation that redox movement improves, not suppresses, capacitation biology	Physiologic-shift responder: improved capacitation proxy with ORP normalization<br>Over-quenching phenotype: reduced capacitation proxy despite lower ORP	Immediate review if capacitation proxy deteriorates after therapy initiation, especially with concurrent fall in ORP/reducing-tone rise
**Clinical reproductive endpoints**	Predefine whether cohort is natural conception vs ART<br>Record female partner age and key female factors (to avoid misattribution)	Track through at least one attempt window (e.g., 3–6 months for natural conception; one ART cycle if applicable)	For natural conception: ongoing pregnancy (and, if feasible, live birth)<br>For ART: fertilization rate (ICSI), usable blastocyst rate, miscarriage<br>The manuscript highlights the need to avoid 'semen-only' surrogates	Clinically concordant responder: mechanistic gains (ORP/SDF/capacitation) accompanied by improved clinical outcome probability	Stop/modify if no mechanistic improvement by a prespecified interim look (e.g., 6 weeks) and patient is at risk of delay (advanced maternal age)

Domain	Minimum reporting elements (baseline)	Intervention monitoring (timing)	Endpoints (mechanistic + clinical)	Response categories (predefined)	Safety/stop criteria (avoid RS overshoot)
**Quality control & reporting**	Assay reproducibility, intra-/inter-assay coefficient of variation where available; standardized abstinence; sample handling (time/temperature); operator training	Ongoing adherence checks; adverse event reporting; supplement accountability	Transparent reporting to allow synthesis across trials (reduces meta-analytic heterogeneity noted in the manuscript)	N/A	N/A
